# Patients With Cancer Searching for Cancer- or Health-Specific Web-Based Information: Performance Test Analysis

**DOI:** 10.2196/23367

**Published:** 2021-08-16

**Authors:** Lukas Lange-Drenth, Holger Schulz, Gero Endsin, Christiane Bleich

**Affiliations:** 1 Department of Medical Psychology University Medical Center Hamburg-Eppendorf Hamburg Germany; 2 VAMED Rehabilitation Clinic Lehmrade Lehmrade Germany

**Keywords:** telemedicine, eHealth, eHealth literacy, digital literacy, internet, web-based, health information, health education, cancer, mobile phone

## Abstract

**Background:**

Searching the internet for cancer-related information helps patients with cancer satisfy their unmet information needs and empowers them to play a more active role in the management of their disease. However, to benefit from the search, patients need a sufficient level of skill to search, select, appraise, and apply web-based health information.

**Objective:**

We aim to study the operational, navigational, information, and evaluation skills and problems of patients with cancer performing cancer-related search tasks using the internet.

**Methods:**

A total of 21 patients with cancer were recruited during their stay at the rehabilitation clinic for oncological rehabilitation. Participants performed eight cancer-related search tasks using the internet. The participants were asked to think aloud while performing the tasks, and the screen activities were recorded. The types and frequencies of performance problems were identified and coded into categories following an inductive coding process. In addition, the performance and strategic characteristics of task execution were summarized descriptively.

**Results:**

All participants experienced problems or difficulties in executing the tasks, and a substantial percentage of tasks (57/142, 40.1%) could not be completed successfully. The participants’ performance problems were coded into four categories, namely operating the computer and web browser, navigating and orientating, using search strategies, and evaluating the relevance and reliability of web-based information. The most frequent problems occurred in the third and fourth categories. A total of 90% (19/21) of participants used nontask-related search terms or nonspecific search terms. A total of 95% (20/21) of participants did not control for the source or topicality of the information found. In addition, none of the participants verified the information on 1 website with that on another website for each task.

**Conclusions:**

A substantial group of patients with cancer did not have the necessary skills to benefit from cancer-related internet searches. Future interventions are needed to support patients in the development of sufficient internet-searching skills, focusing particularly on information and evaluation skills.

## Introduction

### Background

Searching the internet for cancer-related information enables patients with cancer to satisfy their unmet information needs and empowers them to play a larger role in the management of their disease. Unmet information needs are one of the most frequently reported unmet supportive care needs of patients with cancer (6%-93%) during the treatment and posttreatment phases [1]. Patients with cancer prefer to receive as much information as possible about their disease [2,3]. The most common topics of cancer-related information sought on the web are information regarding the diagnosis, prognosis, disease stage, treatment options, or side effects of treatment [4-6].

The percentage of patients with cancer who use the internet to search for cancer-related information is high. In 1 Swedish, 1 American, and 1 Dutch sample, 63%-75% of the participants used the internet to search for cancer-related information or general health information [4,7,8]. The prevalence will continue to rise in the future owing to the increasing use of the internet worldwide [9].

Patients with cancer have various reasons for searching cancer-related web-based information. They use the internet to develop questions to discuss with their physician, verify information given by their physician, or seek alternative treatments [4]. Moreover, they feel that the amount of information they receive from their physician is insufficient [10].

Searching the internet for cancer-related information is positively associated with patient-reported outcomes and socioeconomic characteristics of patients with cancer. Patients with cancer who search the internet for cancer-related information are more involved in medical decision-making [11], feel better informed about their disease [7], have a higher level of self-reported health [12] and quality of life [13], are more likely to have a partner [8], and are younger and more educated [4,8,13] than patients who do not search the internet. In addition, internet health information seeking can improve the patient-physician relationship of patients with acute or chronic conditions, depending on whether the patients discuss the information with their physicians [14].

Nevertheless, to benefit from cancer-related internet searching, cancer-related web-based information must be reliable, and patients with cancer need a sufficient level of skills to search, select, appraise, and apply web-based health information [15,16]. However, the quality of cancer-related web-based information varies widely [17-23]. Information on websites is often incomplete and does not provide a basis for well-informed medical shared decision-making [17-23]. Only half (52%) of the patients with cancer trust the internet as a source of cancer-related information [24]. A total of 3 previous studies [15,25,26] analyzed essential skills to properly search the internet for health-related information. These 3 studies used performance tests and observed participants while executing health-related search tasks on the internet. The essential skills observed during task execution can be divided into 2 categories. First, people need operational and navigational skills to use a computer and web browser, that is, using a keyboard, mouse, or touch screen; navigating forward and backward between websites; and maintaining orientation on a website [15,25]. Second, they need information and evaluation skills to search, find, and assess web-based information, that is, formulate adequate search terms, choose a relevant search result, or check the source of information [15,25]. The results of the first study indicated that approximately one-third of the participants had severe problems in using operational and navigational skills [25]. Similar to the first study, the sample in the second study had, on average, a sufficient level of these skills [15]. The third study did not evaluate these 2 skills [26]. The levels of information and evaluation skills observed in the 3 studies seemed to be much lower [15,25,26]. Many participants reported problems choosing relevant search terms (14/15, 93%), selecting a reliable search result (13/15, 87%), and not checking the source of information (14/15, 93%) in at least 1 task [25]. Furthermore, none of the participants controlled the source of information, the topicality of the information, or how the information had been compiled [26].

Thus far, research on internet searching skills has focused on general healthy populations [15,26] or patients with rheumatoid arthritis [25]. The internet-searching skills of patients with cancer have not yet been studied.

### Objective

Therefore, the primary goal of this study is to gain insight into the operational, navigational, information, and evaluation skills and problems of patients with cancer performing cancer-related search tasks using the internet.

## Methods

### Study Design

A performance test was conducted to obtain in-depth insight into the operational, navigational, information, and evaluation skills and problems of patients with cancer using the internet to search for cancer-related information on the web. Three qualitative methods of data collection were used: (1) the think-aloud method [27] combined with (2) the study administrator’s *real-time* notes and (3) video and audio data of the participants’ screen activity.

The report of this study followed the recommendations of the Standards for Reporting Qualitative Research, consisting of 21 items that aimed to improve the transparency of all aspects of qualitative research by providing clear standards for reporting qualitative research (Multimedia Appendix 1) [28].

The study protocol for this qualitative study is freely available at the Open Science Framework [29] and was published before the recruitment of the first participant.

### Setting, Participants, and Recruitment

The participants were recruited during the first week of their 3-week stay at a rehabilitation clinic for oncological inpatient rehabilitation. Recruitment was conducted by the medical director (GE) of the clinic who approached the participants during the patient consultations. Patients were included if they had been diagnosed with any type of cancer and if they had sufficient oral and written proficiency in the German language. An appointment for the performance test was scheduled within the following week, and the participants received informed consent forms. Informed consent included information about the study goal, potential risks and benefits of the study, the voluntary nature of participation, and the type and duration of data storage. The participants were instructed to sign the informed consent before data collection. All appointments occurred at the rehabilitation clinic and were conducted by the same researcher (LLD). The sample size in this study was based on the concept of theoretical saturation [30], which is defined as the point when no new information, themes, or topics emerge from the data. Saturation, in the context of this study, indicates that no new performance problems were observed among the participants.

### Procedure and Materials

Each appointment started with a short questionnaire to collect the following data: (1) the participants’ socioeconomic characteristics (age, gender, education, and marital status); (2) their medical characteristics (cancer type, time since cancer diagnosis, and self-perceived health status measured using the second-to-last items of the German version of the EORTC QLQ C-30 [European Organization for Research and Treatment of Cancer Quality of Life Questionnaire-30] [31], with response options ranging from 1=very poor to 7=excellent); and (3) their general and cancer-related internet usage characteristics (internet experience, daily time spent on the internet, internet use for cancer-related topics, frequency of health-related internet use, and self-perceived internet searching skills). The participants had to evaluate their internet searching skills on four 5-point scales (ranging from *very bad* to *very good*) that measured the participants’ self-perceived ergonomic skills, navigational skills, evaluating information reliability skills, and determining relevance skills [16].

Performance tests were started when all the items were completed. The patients executed 8 cancer-related internet search tasks (Textbox 1) based on the most common topics of cancer-related information sought on the web [4,5]. The order of the tasks was randomized for each participant because a learning effect was expected to affect the performance of subsequent tasks. The tasks were pilot tested with 4 patients with cancer to ensure comprehensibility and applicability. The participants of the pilot study were recruited from the Outpatient Clinic for Psycho-Oncology of the University Medical Center Hamburg-Eppendorf. The pilot study contained 10 search tasks. Two tasks were deleted because none of the participants were able to complete these tasks independently.

**Description of the cancer-related internet search tasks.**

**Description of the Cancer-Related Internet Search Tasks**

- Imagine that you have noticed the following effects on your physical and mental well-being during your cancer treatment: listlessness, physical and mental exhaustion that does not improve even by sleep or rest. Search the internet for the symptom’s name.
- Please search the internet for treatments or methods of treatment for chronic or persistent “fatigue” (this task always came after task 1).
- Please search the internet for various providers who offer psycho-oncological counseling in the Hamburg area (postcode: 22529).
- Formulate a disease-related question you have had in the past and show how you would approach this on the internet.
- Please search the patient guidelines of the German Cancer Society for your specific type of cancer.
- Search for the information sheet of the Cancer Information Service “Cancer on the internet: Surf safely.”
- With the help of information from the internet, please name 5 possible side effects or symptoms of the specific cancer therapy (eg, chemotherapy and radiotherapy) that you received.
- With the help of information from the internet, please name possible ways that you could change your diet to promote your well-being or reduce side effects.

The performance tests were recorded using *Open Broadcaster Software* (version 26.1.0), which generated video and audio data. The participants were asked and trained to think aloud while performing the tasks. The verbalization of the participants’ thoughts allowed the researcher to gain insight into the participants’ cognitive processes while searching for web-based information [32]. In addition, the researcher present observed the participants and recorded real-time notes to identify problems with the hardware operation.

Each performance test was conducted using the same hardware (laptop, mouse, and keyboard) with identical settings. The laptop was connected to an active internet connection and was programmed with the 3 most popular web browsers (Internet Explorer, Mozilla Firefox, and Google Chrome). The participants were instructed to choose the web browser with which they had the most experience. All web browsers began with a blank page. To prevent the participants from being influenced by previous participants’ search activities, the web browser was reset after each participant by removing the web browser history and cookies using CCleaner version 5.44 (Avast Software). If the participants were unable to perform the task, help was offered by the researcher present. The participants received €15 (US $17.70) for participating in the study.

### Data Analysis

Statistical analyses were conducted using SPSS Statistics (version 25, IBM SPSS Inc). The participants’ sociodemographic characteristics, medical characteristics, and general and cancer-related internet usage were summarized descriptively.

Video and audio data, as well as the researcher’s real-time notes, were analyzed to (1) identify participants’ performance problems, (2) evaluate the participants’ performance, and (3) identify performance and strategic characteristics of task execution.

To identify participants’ performance problems, the researchers followed an inductive coding process [33]. Participants’ behavior or statements were initially coded and subsequently grouped into categories and subcategories that were then named. The category names were partly based on categories from previous research [25]. The number of problems encountered per task was then determined.

The evaluation of the participants’ performance per task and the difficulty of the tasks were based on two variables: (1) could the participants complete their task *completely independently*, *with help,* or *not able to complete* the task at all, and (2) the time needed to perform the task (the more time needed to complete a task, the higher the difficulty of the task).

The execution and strategic characteristics of task execution were described by six variables: (1) the used web browser (Internet Explorer, Mozilla Firefox, or Google Chrome), (2) the starting point (eg, a specific website or a search engine), (3) the position of the opened website in the Google search listings from top to bottom (the position of the opened website in the search listings is an indicator of whether participants look beyond the first search results), (4) the number of words per search query (the use of a single search term was considered too unspecific; it is more important for the successful completion of a task to use task-related search terms than a large number of search terms), (5) the number of times a search query needed to be adjusted (a higher number of adjustments per task indicated a higher difficulty of the task), and (6) the name of the opened websites (Do the most often opened websites have a good content ranking and a commercial interest?) [21].

The influence of the participants’ education (>10 years of school education vs ≤10 years of school education), age (above vs below median), self-perceived internet skills (above vs below median), internet experience (above vs below median), and time since cancer diagnosis (above vs below median) on the participants’ average number of problems per task and percentage of successfully completed tasks were analyzed using 2-tailed *t* tests for independent groups. For additional interpretation, effect sizes were calculated: the values of Cohen *d* for small, medium, and large effects were 0.2, 0.4, and 0.8, respectively [34]. The α level of significance was set at α=.05.

### Ethics Statement

The study was conducted in accordance with the Code of Ethics of the Declaration of Helsinki and was surveyed by the Ethics Committee of the Medical Association (Hamburg, Germany). Written informed consent was obtained from all the participants before participation.

## Results

### Participants’ Characteristics and Participants’ Internet Use

Slightly more women (12/22, 55%) than men participated in the study (Table 1). The participants’ ages ranged between 25 and 81 years (mean 57 years, SD 11.9 years). Almost three-fourths of the participants lived with a partner (16/22, 73%). Most (13/22, 59%) of the sample had 10 years or less of schooling, whereas 27% (6/22) had a university degree. Breast (6/22, 27%), colon (4/22, 18%), and prostate cancer (3/22, 13%) were the most frequently reported cancer diagnoses. The participants received their diagnosis, on average, 28 (SD 57.8) months prior. The average self-perceived health status score was 4.5.

**Table 1:** Medical and sociodemographic characteristics of the participants (N=22).

Participant characteristics			Values
Age (years), mean (SD; range)			56.8 (12; 25-81)
Gender (female), n (%)			12 (55)
**Marital status, n (%)**			
	Living alone	6 (27)	
	Living with a partner	16 (73)	
**Highest educational achievement, n (%)**			
	University degree	6 (27)	
	13 years of school education	3 (14)	
	10 years of school education	9 (41)	
	9 years of school education	4 (18)	
**Cancer type, n (%)^a^**			
	Breast cancer	6 (27)	
	Colon cancer	4 (18)	
	Prostate cancer	3 (13)	
	Lung cancer	2 (9)	
	Kidney cancer	2 (9)	
	Other	8 (32)	
Time since cancer diagnosis (months), median (range)			6 (1-207)
Self-perceived health status, mean (SD)			4.5 (1.0)

^a^Multiple selection.

The participants’ mean internet experience was 15 years (Table 2). Most of the participants (13/22, 59%) used the internet for less than 1 hour per day. The most common cancer-related activities on the internet were searching for cancer-related information (14/22, 64%) and communication with relatives (14/22, 64%). More than half (14/22, 64%) of the participants used the internet less than once a month for health care reasons. The participants rated their ergonomic skills, evaluating information reliability skills, navigation skills, and determining information relevance skills as *medium* to *good*.

**Table 2:** General and cancer-related internet usage of participants (N=22).

Participant characteristics				Values
Internet experience (years), mean (SD; range)				15.4 (7.7; 0-30)
**Daily time spent on the internet (minutes), n (%)**				
	No utilization			1 (5)
	0-30			5 (23)
	30-60			7 (32)
	60-120			6 (27)
	>120			3 (15)
**Types of internet use for cancer-related topics, n (%)^a^**				
	Obtaining general information about my cancer (ie, treatment information)			14 (64)
	Communication with relatives or friends			14 (64)
	Search for treatment options			12 (55)
	Search for health care professionals			8 (36)
	Verifying information received from health care professionals			7 (32)
	Contact health care professionals (ie, oncologist)			4 (18)
	Contact pharmacist			3 (14)
	Contact other patients			3 (14)
	Search for alternative treatment options			3 (14)
	Search scientific data (ie, Google Scholar)			3 (14)
**Frequency of internet use as a part of health care, n (%)**				
	Never			2 (9)
	Rarely			12 (54)
	More than once a month			3 (14)
	More than once a week			4 (18)
	Daily			1 (5)
**Self-perceived internet-searching skills**				
	Value, range			1-5
	**Different self-perceived internet-searching skills, mean (SD)**			
		Ergonomic skills	3.5 (1.4)	
		Navigation skills	3.2 (1.3)	
		Evaluating information reliability skills	3.5 (0.7)	
		Determining information relevance skills	3.5 (0.8)	

^a^Multiple selection.

### Execution of Cancer-Related Tasks and Problems Encountered

#### Search Strategy and Effectiveness of Searches

Performance tests of the 21 participants were included in the analysis. The performance of participant 22 was excluded because the participant could not execute the tasks due to stress. On average, the participants executed 6.8 tasks. Participants 1 and 21 performed only 2 tasks. A total of 57% (12/21) of participants executed all 8 tasks. All data on task execution and performance problems are available in Figshare [35].

None of the participants used medical websites as a starting point. All search tasks were started using the Google search engine. On average, the participants successfully completed 59.9% (85/142) of all the tasks. Task F (Search the information sheet of the Cancer Information Service) had the highest rate of successful completions (14/18, 78%) and took participants, on average, the shortest time to execute (Table 3). Task E (Search the patient guideline for your cancer type) had the lowest rate of completion (6/19, 32%). The longest mean time (mean 323 seconds) to execute a task was observed for task D (retrieve previously searched disease information). A total of 86% (18/21%) of participants used the same web browser for all the tasks. Google Chrome was used in 80.2% (114/142) of search queries. The participants opened 192 webpages (the same website was counted every time it was opened) from 61 different websites during their 142 search queries (Multimedia Appendix 2). The 2 most frequently opened websites were provided by professional associations with generally good content rankings [21]. The participants usually selected one of the first websites from Google search listings. Of the 192 opened webpages, 163 (84.9%) webpages were ranked among the first 5 Google search results. Websites from the second page of Google search listings were opened 4 times (4/192, 2.1%). On average, the participants used 4.2 search terms per task. A single search term was used for 4.9% (7/142) of search queries. In addition, in 27.5% (39/142) of search queries, the participants decided to adjust the search terms to improve the Google search results.

**Table 3:** Performance and strategic characteristics of the task execution (n=21).

Tasks			A^a^ (n=19)		B^b^ (n=17)		C^c^ (n=17)		D^d^ (n=17)		E^e^ (n=19)		F^f^ (n=18)		G^g^ (n=17)		H^h^ (n=18)
**Task completion, n (%)**																	
	Completed independently	12 (63)		10 (59)		10 (59)		11 (65)		6 (32)		14 (78)		12 (71)		10 (55)	
	Completed with help	1 (5)		2 (12)		0 (0)		0 (0)		0 (0)		1 (6)		0 (0)		1 (6)	
	Not completed	6 (32)		5 (29)		7 (41)		6 (35)		13 (68)		3 (16)		5 (29)		7 (39)	
**Time per task (seconds)**																	
	Value, mean (SD; range)	195 (121; 58-461)		220 (105; 68-467)		253 (207; 81-843)		323 (188; 116-696)		209 (117; 63-411)		177 (177; 44-746)		228 (146; 36-529)		235 (149; 91-489)	
**Web browser used, n (%)**																	
	Google Chrome	16 (84)		15 (87)		13 (76)		13 (76)		16 (84)		14 (78)		14 (82)		13 (72)	
	Mozilla Firefox	3 (16)		2 (13)		4 (24)		4 (24)		3 (16)		3 (16)		3 (18)		3 (17)	
	Internet Explorer	0 (0)		0 (0)		0 (0)		0 (0)		0 (0)		1 (6)		0 (0)		2 (11)	
**Position of website^i,j^, n (%)**																	
	First search result	11 (41)		11 (42)		6 (27)		9 (36)		15 (63)		12 (57)		7 (33)		5 (19)	
	Second to fifth search result	13 (48)		14 (54)		9 (41)		8 (32)		9 (37)		9 (43)		10 (48)		15 (58)	
	Sixth to tenth search result	3 (11)		1 (4)		7 (32)		8 (32)		0 (0)		0 (0)		4 (19)		2 (8)	
	Second page of Google listings	0 (0)		0 (0)		0 (0)		0 (0)		0 (0)		0 (0)		0 (0)		4 (15)	
**Words per search query**																	
	Value, mean (SD)	5.6 (4.7)		3.3 (2.2)		4.8 (3.5)		3.4 (2.3)		2.8 (0.6)		3.8 (1.6)		3.4 (1.2)		3.9 (2.8)	
	Single search term, n (%)	0 (0)		2 (8)		1 (5)		2 (8)		0 (0)		2 (10)		0 (0)		0 (0)	
**Adjustments of a search query, n (%)**																	
	Amount	6 (22)		2 (8)		5 (23)		6 (24)		4 (17)		6 (29)		6 (29)		4 (15)	

^a^Identify the symptom fatigue.

^b^Search treatment methods for cancer-related fatigue.

^c^Search service providers who offer psycho-oncological counseling.

^d^Retrieve previously searched disease information.

^e^Search the patient guideline for your cancer type.

^f^Search the information sheet of the Cancer Information Service.

^g^Search for five symptoms of the treatment you received.

^h^Search for options to change your diet.

^i^Position of opened websites in the Google search listings from top to bottom.

^j^In some cases, participants opened more than one website. The position in the Google search listings of each website is listed here.

The participants’ performance problems were coded into four categories concerning internet searching skills (Table 4): (1) operating the computer and web browser, (2) navigating and orientating, (3) using search strategies, and (4) evaluating the relevance and reliability of web-based information. In addition, problems in understanding the task and focusing on the task were observed.

**Table 4:** Performance problems and number of participants experiencing those problems for each task.

Tasks		A^a^ (n=19)	B^b^ (n=17)	C^c^ (n=17)	D^d^ (n=17)	E^e^ (n=19)	F^f^ (n=18)	G^g^ (n=17)	H^h^ (n=18)	Total^i^ (n=21)
**Operating the computer or web browser, n (%)**										
	Operating the keyboard	5 (26)	4 (24)	3 (18)	3 (18)	5 (26)	4 (22)	4 (24)	4 (22)	7 (33)
	Controlling the mouse	6 (32)	2 (12)	2 (12)	2 (12)	5 (26)	5 (28)	3 (18)	6 (33)	8 (38)
	Using the scroll bar	0 (0)	0 (0)	0 (0)	0 (0)	2 (11)	1 (6)	0 (0)	0 (0)	3 (14)
	Operating the web browser	2 (11)	1 (6)	3 (18)	3 (18)	0 (0)	1 (6)	3 (18)	3 (17)	11 (48)
	Reading difficulties	0 (0)	1 (6)	0 (0)	0 (0)	0 (0)	0 (0)	1 (6)	0 (0)	2 (10)
	Participants with >1 problem per task	5 (26)	0 (0)	2 (12)	3 (18)	3 (16)	3 (18)	2 (12)	4 (22)	6 (29)
**Navigating and orientating, n (%)**										
	Keeping orientation on a website	0 (0)	1 (6)	0 (0)	0 (0)	1 (6)	1 (6)	1 (6)	2 (11)	5 (29)
	Using and understanding a PDF file	1 (5)	0 (0)	0 (0)	0 (0)	3 (11)	0 (0)	0 (0)	0 (0)	3 (14)
	Using dropdown lists	0 (0)	1 (6)	0 (0)	0 (0)	1 (6)	0 (0)	0 (0)	0 (0)	2 (10)
	Orientation in the Google search engine	1 (5)	0 (0)	0 (0)	0 (0)	0 (0)	0 (0)	0 (0)	0 (0)	1 (5)
	Participants with >1 problem per task	0 (0)	0 (0)	0 (0)	0 (0)	0 (0)	1 (6)	1 (6)	1 (6)	2 (10)
**Using search strategies, n (%)**										
	Too broad search query	0 (0)	2 (12)	1 (6)	2 (12)	0 (0)	2 (12)	0 (0)	0 (0)	5 (24)
	Nonspecific or nontask-related search query	9 (47)	5 (29)	9 (53)	1 (6)	5 (26)	4 (22)	4 (24)	2 (11)	19 (90)
	Spelling and grammatical errors in search query	2 (11)	1 (6)	5 (29)	2 (12)	5 (26)	4 (22)	2 (12)	0 (0)	11 (52)
	Adjusting the search query	1 (5)	0 (0)	1 (6)	0 (0)	2 (11)	0 (0)	1 (6)	1 (6)	6 (29)
	Selection of task-related search results	0 (0)	0 (0)	3 (18)	2 (12)	0 (0)	4 (22)	4 (24)	3 (16)	11 (52)
	Selecting physician instead of patient guidelines	N/A^j,k^	N/A^k^	N/A^k^	N/A^k^	8 (42)	N/A^k^	N/A^k^	N/A^k^	N/A^k^
	Participants with >1 problem per task	3 (16)	0 (0)	5 (29)	0 (0)	5 (26)	2 (12)	1 (6)	1 (6)	9 (43)
**Evaluating relevance and reliability, n (%)**										
	Controlling the source of information	19 (100)	17 (100)	17 (100)	17 (100)	18 (95)	18 (100)	17 (100)	17 (94)	21 (100)
	Searching in commercial websites	1 (5)	0 (0)	2 (12)	2 (12)	0 (0)	0 (0)	3 (18)	6 (33)	8 (38)
	Verifying the information	17 (89)	15 (88)	16 (94)	16 (94)	N/A^l^	N/A^l^	14 (82)	17 (94)	21 (100)
	Scanning a website for relevant information	2 (11)	1 (6)	0 (0)	0 (0)	0 (0)	1 (6)	0 (0)	5 (28)	6 (29)
	Participants with >1 problem per task	17 (89)	15 (88)	15 (88)	16 (94)	0 (0)	1 (6)	15 (88)	18 (100)	21 (100)
**Understanding of the task and keeping focus, n (%)**										
	Understanding the task	4 (21)	2 (12)	0 (0)	0 (0)	1 (5)	2 (12)	3 (18)	0 (0)	7 (33)
	Forgetting the task	0 (0)	2 (12)	1 (6)	1 (6)	3 (16)	3 (17)	0 (0)	0 (0)	9 (43)
	Keeping focus	2 (11)	1 (6)	1 (6)	3 (18)	0 (0)	1 (6)	1 (6)	0 (0)	5 (24)

^a^Identify the symptom fatigue.

^b^Search treatment methods for cancer-related fatigue.

^c^Search service providers who offer psycho-oncological counseling.

^d^Retrieve previously searched disease information.

^e^Search the patient guideline for your cancer type.

^f^Search for the information sheet of the Cancer Information Service.

^g^Search for five symptoms of the treatment you received.

^h^Search for options to change your diet.

^i^Number of participants who experienced this problem during the execution of at least one task.

^j^N/A: not applicable.

^k^The problem was task-specific (task 5).

^l^Verifying the information was not applicable for this task because participants were instructed to search for a particular website.

#### Operating the Computer and Web Browser

A total of 62% (13/21) of participants had at least 1 problem using computer hardware (keyboard and mouse) or problems using basic web browser functions.

In total, 33% (7/21) of participants had problems using the keyboard, especially locating keys while typing search terms or searching for the *enter* key. In addition, 38% (8/21) of participants had problems controlling the mouse, mainly the double-clicking of icons (eg, web browser). A total of 14% (3/21) of participants experienced difficulties with the scroll bar. A total of 52% (11/21) of participants had problems operating the web browser. Frequent problems included finding the web browser icon on the desktop and closing and reopening the web browser to adjust the search terms instead of using the web browser’s *back* button. Furthermore, 10% (2/21) of participants had problems reading the text of a website because of the small font size.

Overall, the operational problems were not task-specific. Most of the operating problems (94/122, 77%) were experienced by the same 6 participants. After completing several tasks, the present researcher observed that the participants became increasingly frustrated with the recurring operational problems.

In addition, 2 behaviors were observed but were not coded as operational problems. A total of 81% (17/21) of participants closed the web browser after every individual task and reopened the browser for the next task. Furthermore, 71% (15/21) of participants used a single tab for all searches.

#### Navigating and Orienting

A total of 33% (7/21) of participants experienced at least one problem with navigation and orientation in web browsers and on websites. Problems often occurred when the websites had complex structures, such as different graphical control elements (ie, dropdown lists or anchor links).

A total of 24% (5/21) of participants had problems maintaining their orientation on a website. They lost their orientation for various reasons. Twice, the participants felt confused by the browser’s starting page (eg, “This is not where I wanted to be” [Participant 8]). Two other times, the participants did not understand the function of the anchor link at the top of the website, which would have helped them jump to the relevant information in the text. A total of 14% (3/21) of participants could not distinguish an opened PDF file from a website. In total, 10% (2/21) of participants did not find relevant information on websites because they could not use the websites’ dropdown lists. One participant lost orientation due to the Google option *related searches* (eg, “I did not write that” [Participant 8]).

Notably, most of the orientation and navigation problems (94/122, 77%) were experienced by the same group of 6 participants who encountered most of the operational problems.

#### Using Search Strategies

A total of 95% (20/21) of participants experienced at least 1 problem using search strategies. Most of the problems occurred in the first stage when the participants formulated the search terms.

A total of 24% (5/21) of participants used only single search terms that were too broad to successfully complete the tasks. In total, 90% (19/21) of participants used nontask-related search terms or nonspecific search terms. For example, Participant 5 used the search terms *patient Hamburg* to find the patient guidelines of the German Cancer Society (task 5; nontask-related). Participant 11 searched for *psychological support* instead of *psycho-oncological support* (task 3; unspecific search). In total, 52% (11/21) of participants made spelling or grammatical errors in their search queries. These participants (9/11, 81%) usually did not adapt the search terms, and the Google option *did you mean* was not used to correct the errors.

The use of nontask-related, nonspecific, or grammatically incorrect search terms made the participants adjust their search terms or select a nontask-related webpage from Google search listings. A total of 29% (6/21) of participants experienced problems adjusting the search terms. For example, Participant 9 adjusted the original search query from *side effect of cancer therapy* to *side effect of cancer*, a query that was still not task-related (task 1: identify the symptom fatigue). A total of 52% (11/21) of participants selected nontask-related websites from the search listings. Many of these participants (5/11, 45%) randomly opened one of the first search results (“I am going on a random website; usually I don’t like to pick the first website” [Participant 14]; task 2). They did not look at the URL or Google snippet (short description of the website’s content) to be informed about the website’s content.

To complete task 5, the participants had to find the patient guidelines (PDF file) of their specific cancer type. A total of 38% (8/21) of participants selected clinical practice guidelines instead of the patient guidelines. Both types of guidelines can be found on the same website.

#### Evaluating Relevance and Reliability

All participants experienced at least 1 problem while evaluating their relevance and reliability. None of the participants controlled the source or topicality of the information, except for Participant 19. Furthermore, none of the participants verified the information on 1 website with that on another website for each task. Most of the participants only opened a second website when they were not satisfied with the information on the first website. A total of 19% (4/21) participants made critical comments regarding the reliability of commercial websites; for example:

The first search result is an advertisement. Therefore, I will not consider that webpage.

Participant 7

Nevertheless, 38% (8/21) of participants selected and searched the providers’ websites with a commercial interest. In total, 4 of these participants even opened websites marked as ads by Google. A total of 29% (6/21) of participants did not scan the selected websites for relevant information to complete the task. They read the websites’ headings and then completed the task because they were convinced that the information they were looking for could be found on the website.

Notably, 71% (15/21) of participants made comments regarding the following: (1) the reliability of certain websites; for example:

I got offers. Here, from yelp.com. I have problems with opening this website because for me that is dubious information.

Participant 17

(2) the reliability of certain types of websites; for example:

What I would not read are patient forums. Where some laymen write what they did...I would rely on medical tips.

Participant 2

or (3) the internet as a source of cancer-related information; for example:

The internet in general is way too superficial. I read a book to gather information about that.

Participant 4

#### Understanding the Task and Staying Focused

A total of 33% (7/21) of participants experienced problems understanding the tasks (“I don’t know what I am supposed to search” [Participant 14]; task 1), usually (10/12, 83%) resulting in nontask-related search queries. A total of 43% (9/21) of participants forgot the task during execution and had to reread it. A total of 24% (5/21) of participants were distracted by other nontask-related information. For example, Participant 3 became distracted by a brochure about *sexuality and cancer* while executing task 6.

### Relationship Between Patient Characteristics and Performance Parameters

Participants 1 and 21 were not included in this analysis because they executed fewer than 3 tasks. The participants who were younger (mean 2.7, SD 1.0) and had higher self-perceived internet skills (mean 2.9, SD 1.1), on average, encountered significantly fewer (*t_17_*=−2.78, *P*=.01, Cohen *d*=1.30; *t_17_*=−2.33, *P*=.03, Cohen *d*=1.07) performance problems per task than those who were older (mean 4.6, SD 1.8) and had lower self-perceived internet skills (mean 4.5, SD 1.8; Multimedia Appendix 3). Both the differences had a large effect size. In addition, participants with higher self-perceived internet skills (mean 75.6, SD 18.1) completed a significantly higher percentage of tasks successfully (*t_17_*=2.65; *P*=.02; Cohen *d*=1.23) than those with lower self-perceived internet skills (mean 44.9, SD 30.2). In addition, differences with a large, medium, and small effect size can be found: (1) younger participants completed a higher percentage of tasks successfully (Cohen *d*=0.87); (2) participants with more internet experience completed a higher percentage of tasks successfully (Cohen *d*=0.87) and had fewer performance problems (Cohen *d*=0.66) than patients with less experience; (3) participants with higher education completed a higher percentage of tasks successfully (Cohen *d*=0.46) and had fewer performance problems (Cohen *d*=0.40) than participants with a lower education; and (4) participants who had more time since diagnosis completed a higher percentage of tasks successfully (Cohen *d*=0.30) and had fewer performance problems (Cohen *d*=0.40) than participants with a lower education.

## Discussion

### Principal Findings

This study examined the level of operational, navigational, information, and evaluation skills of a sample of patients with cancer performing 8 cancer-related search tasks using the internet. The results indicate that a substantial group of patients with cancer did not have the necessary operational, navigational, information, and evaluation skills to benefit from cancer-related internet searches. A total of 29% (6/21) of participants had major problems with the operation of the hardware, operation of the computer and web browser, and with navigation and orientation in web browsers and on websites. These participants produced three-fourths (94/122, 77%) of the operational and navigational problems. These problems caused great frustration among the participants and often resulted in tasks not being completed successfully. A total of 6 participants completed only 29% (12/42) of their tasks successfully. Although the operational and navigational skills of most participants (15/21, 71%) seemed to be sufficient for searching the internet, the information and especially the evaluation skills were much lower. Many participants struggled with formulating a task-related search query (19/21, 90%), selecting a task-related search result (11/21, 52%) of a provider without a commercial interest (8/21, 38%), and browsing the website to find the answer to the task (6/21, 29%). Strikingly, only 19% (4/21) of participants verified the information on 1 website with that on another website, and only 5% (1/21) of participant informed himself about the provider of the website. The remaining participants seemed to take no interest in the source or topicality of the information. These findings are alarming because previous research has shown that the quality of cancer-related web-based information varies widely [17-23].

### Comparison With Previous Work

Our results are consistent with those of previous studies that used performance tests to analyze the internet-searching skills of healthy participants [15,26] and patients with rheumatoid arthritis [25]. Operational and navigational problems occurred in only approximately one-third of the samples [15,25]. In addition, almost all the participants of this study and three similar studies had problems with information and evaluation skills [15,25,26]. Owing to the similarity of the results, we believe that the identified internet searching problems can also be found in patients with other health conditions and in the healthy population. In addition to the National Action Plans that plan to increase the health literacy of the entire population in the future [36,37], further web-based interventions are needed to increase the internet-searching skills of patients with low skills presently [38-40].

The rate of search queries with single search terms was the main difference between the search strategies used in 2002 [26] and those used in this study. In 2002, 65% of all search queries comprised a single search term [26] compared with 4.9% (7/142) in this study. The longer average internet experience of our sample (15.4 years vs 2.5 years) could be a possible explanation for choosing to use more search terms. However, information skills did not seem to grow with years of internet experience [41].

An exploratory analysis of our data indicated that younger age, higher self-perceived internet skills, more internet experience, and higher education were associated with encountering fewer performance problems and completing a higher percentage of tasks successfully. In addition, more time since diagnosis was associated with fewer performance problems and a slightly higher percentage of successfully completed tasks. The results of our exploratory analysis should be interpreted carefully because our sample size (n=19) was too small to make assumptions about the population of patients with cancer. However, previous research on internet-searching skills using performance tests confirmed that younger age, a higher level of education, more internet experience, and higher self-perceived internet skills are associated with more successful task completion [25,41]. Younger participants have higher operational and navigational skills but particularly poor performance regarding evaluation skills [41,42]. Education and self-perceived internet skills are associated with operational, navigational, information, and evaluation skills, whereas internet experience only has a positive influence on operational and navigational skills [41]. In addition, we analyzed the influence of time since cancer diagnosis on the participants’ test performance, assuming that patients who had more time looking for cancer-related information on the web may know of more reliable providers of web-based cancer information than patients who recently received their diagnosis. A possible explanation for the lack of a stronger association with participants’ performance might be that having more time since diagnosis may only be associated with patients’ evaluation skills but not their operational, navigational, and information skills. Future performance tests with larger sample sizes are needed to examine this question.

### Limitations

This study had several limitations. First, the participants performed the tasks in an artificial research setting under experimental conditions. They may have felt more nervous than if they had been in a natural setting. For example, Participant 22 did not start the performance test because of stress. In addition, the participants may have felt time pressure to perform the tasks and may have focused less on evaluating the reliability or topicality of the websites. We tried to minimize the pressure to perform and time pressure by explicitly reminding participants to take their time and that there were no right or wrong answers. Second, some participants had to use unfamiliar hardware as they accessed the internet exclusively with their smartphones or tablets [9]. This may have increased the number of operational problems experienced. Asking the participants what type of digital device they use to access the internet would have helped to distinguish among the users. Future performance studies should also consider enabling participants to perform tasks on a smartphone or tablet. Third, to ensure that the tasks were related to the interests and needs of patients with cancer, we formulated search tasks that covered the most common topics of cancer-related information sought on the web [4,5] and pilot-tested the tasks. However, compared with a real setting, answering our cancer-related questions had no direct effect on the treatment or well-being of the participants. Therefore, the participants may have been less motivated to evaluate the website’s reliability or to verify the information found on a second website. Fourth, the study sample was small and most likely nonrepresentative. No national or international studies with representative samples have been conducted yet that allow statements on the internet searching skills of entire population groups or patients with cancer [43]. However, we expect that our sample had higher skills than the population of patients with cancer for the following three reasons: (1) the participants volunteered to participate in the study; (2) the participants’ average internet experience in years (mean 15.4 years) was high, and operational and navigational skills grew with years of internet experience [41]; and (3) the participants were often highly educated (28% had a university degree), with a high level of education being related to high internet searching skills [41]. Future performance test studies should concentrate on older, lesser-educated patients with cancer with little internet experience. Additional important performance problems may be identified because these patient characteristics are associated with low internet-searching skills [25,41]. Fifth, using the concept of data saturation [30] also captures the risk of missing additional important performance problems [44]*.* Previous studies have indicated that even if no new concepts emerge, the possibility of further uncovered concepts in the population cannot be excluded [45,46]. We cannot completely exclude the possibility that we missed certain internet searching problems. Nevertheless, by giving the participants sufficient time to express all of their thoughts and the similarity between our results and those of previous performance tests [15,25], we are convinced that we have identified most of the important problems of patients with cancer using the internet to search for cancer-related information on the web.

### Conclusions

A substantial group of patients with cancer did not have the necessary skills to benefit from cancer-related internet searches. The problems included operating the hardware, navigation and orientation in web browsers and on websites, and in particular formulating a task-related search query and critically evaluating and verifying web-based content. Given the high number of participants with higher education and relatively high internet experience, the need for future interventions or programs to increase the internet-searching skills of patients with cancer may be underestimated in this study. Additional important performance problems may be identified in future studies that concentrate on older, low-educated patients with little internet experience.

## Appendix

Multimedia Appendix 1 Standards for Reporting Qualitative Research checklist.

Multimedia Appendix 2 Names of websites and the number of times they were opened.

Multimedia Appendix 3 Completed tasks and number of encountered problems per assignment related to education level, age, self-perceived internet skills, internet experience, and time since diagnosis.

## Abbreviations

EORTC QLQ C-30: European Organization for Research and Treatment of Cancer Quality of Life Questionnaire-30
