# Supplementary material for: Patients With Cancer Searching for Cancer- or Health-Specific Web-Based Information: Performance Test Analysis
Source: J Med Internet Res. 2021 Aug 16;23(8):e23367. doi: 10.2196/23367 (PMC8406111; doi:10.2196/23367)
Supplement: Multimedia Appendix 2 [file jmir_v23i8e23367_app2.docx]

Table 5. Names of websites and the amount of times they were opened.

| Name of websites | Number of times the website was opened |
| --- | --- |
|  |  |
| krebsinformationsdienst | 46 |
| krebsgesellschaft.de | 31 |
| leitlinienprogramm-onkologie.de | 19 |
| netdoktor.de | 12 |
| deutsche-fatigue-gesellschaft.de | 8 |
| apothekenumschau.de | 6 |
| awmf.de | 3 |
| uke.de | 3 |
| onkologie.hexal.de | 2 |
| wikipedia.de | 2 |
| gesund24.at | 2 |
| zentrum-der-gesundheit.de | 2 |
| derwesten.de | 2 |
| roche.de | 2 |
| ugb.de | 2 |
| zapmeta.de | 2 |
| shop.krebsliga.ch | 2 |
| uni-ulm.de | 2 |
| gesundheitswissen.de | 1 |
| zm-online.de | 1 |
| krebshilfe-wien.at | 1 |
| google.scholar.com | 1 |
| ndr.de/ratgeber | 1 |
| bibliomed-pflege.de | 1 |
| fatigue.info.de | 1 |
| msd-gesundheit.de | 1 |
| informationvine.de | 1 |
| krebs-selbsthilfegruppe.de | 1 |
| careacross.de | 1 |
| kensaq.com | 1 |
| elbkliniken.de | 1 |
| tabea-fachklinik.de | 1 |
| krebshamburg.de | 1 |
| sanego.de | 1 |
| therapie.de | 1 |
| michaelasimon.de | 1 |
| google.maps.com | 1 |
| doktorweigel.de | 1 |
| biokrebs.de | 1 |
| tumorzentrum-muenchen.de | 1 |
| info-blasenkrebs.de | 1 |
| krebsratgeber.de | 1 |
| haema-cbf.charite.de | 1 |
| springer-medizin.de | 1 |
| klinikum.uni-heidelberg.de | 1 |
| viomedo | 1 |
| team-andro.de | 1 |
| frauenmedizin-schaefflerhof.de | 1 |
| senelogie.de | 1 |
| docplayer.org | 1 |
| thieme.de | 1 |
| lymphome.de | 1 |
| gesundheitsinformation.de | 1 |
| leben-mit-brustkrebs.de | 1 |
| prostatakrebs.bps.de | 1 |
| volkskrankheiten.net | 1 |
| forumgesundheit.at | 1 |
| nutricia.de | 1 |
| ratgeber-darmgesundheit.de | 1 |
| gynecology-guide.com | 1 |
| coloplast.de | 1 |
