# Supplementary material for: Patients With Cancer Searching for Cancer- or Health-Specific Web-Based Information: Performance Test Analysis
Source: J Med Internet Res. 2021 Aug 16;23(8):e23367. doi: 10.2196/23367 (PMC8406111; doi:10.2196/23367)
Supplement: Multimedia Appendix 3 [file jmir_v23i8e23367_app3.docx]

Table 6. Completed tasks and number of encountered problems per assignment related to education level, age, self-perceived internet skills, internet experience, and time since diagnosis (8 tasks) (n = 19)

| Participant Characteristics |  | Percentage of successfully completed tasks | Average number of problems per task |
| --- | --- | --- | --- |
|  |  | Mean (SD) | Mean (SD) |
|  |  |  |  |
| **Education** |  |  |  |
|  | > 10 years of education (n = 7) | 68.2 (32.6) | 3.3 (1.9) |
|  | ≤ 10 years of education (n = 12) | 54.4 (27.1) | 4.0 (1.6) |
|  | Group difference^a^: High vs. low education | t = 0.99; p = .34; d = .46 | t = -.87; p = .39;  d = .40 |
| **Age** |  |  |  |
|  | Younger than the median value (n= 9) | 71.8 (22.9) | 2.7 (1.0) |
|  | Older than the median value (n = 10) | 48.4 (30.8) | 4.6 (1.8) |
|  | Group difference^a^: Younger vs. older participants | t =1.85; p = .08; d = .87 | t = -2.78; p = .01; d = 1.30 |
| **Self-perceived internet skills** |  |  |  |
|  | Self-perceived internet skills above the median value (n = 9) | 75.6 (18.1) | 2.9 (1.1) |
|  | Self-perceived internet skills below the median value (n = 10) | 44.9 (30.2) | 4.5 (1.8) |
|  | Group difference^a^: High vs. low self-perceived internet skills | t = 2.65; p = .02; d = 1.23 | t = -2.33; p = .03; d = 1.07 |
| **Internet experience** |  |  |  |
|  | More internet experience than the median value (n = 10 ) | 70.9 (22.0) | 3.2 (1.4) |
|  | Less internet experience than the median value (n = 9) | 46.8 (32.0) | 4.3 (1.9) |
|  | Group difference^a^: More vs. less internet experience | t = 1.92; p = .07; d = 0.87 | t = -1.40; p = .18; d = 0.66 |
| **Time since cancer diagnosis** |  |  |  |
|  | Time since cancer diagnosis above the median (n = 10) | 64.9 (29.1) | 3.3 (1.5) |
|  | Time since cancer diagnosis below the median (n = 9) | 55.9 (30.6) | 4.0 (2.0) |
|  | Group difference^a^: More time vs. less time since cancer diagnosis | t = 0.64; p = .53; d = 0.30 | t = -0.88; p = .39; d = 0.40 |

^a^The group differences are displayed as follows: (1) t-test score; (2) significance level; (3) effect size (Cohen’s d)
